# Molecular Detection and Genotyping of *Enterocytozoon bieneusi* in Black Goats (*Capra hircus*) in Yunnan Province, Southwestern China

**DOI:** 10.3390/ani11123387

**Published:** 2021-11-26

**Authors:** Shi-Chen Xie, Yang Zou, Zhao Li, Jian-Fa Yang, Xing-Quan Zhu, Feng-Cai Zou

**Affiliations:** 1 Key Laboratory of Veterinary Public Health of Yunnan Province, College of Veterinary Medicine, Yunnan Agricultural University, Kunming 650201, China; xieshichen221@163.com (S.-C.X.); xingquanzhu1@hotmail.com (X.-Q.Z.); 2 State Key Laboratory of Veterinary Etiological Biology, Key Laboratory of Veterinary Parasitology of Gansu Province, Lanzhou Veterinary Research Institute, Chinese Academy of Agricultural Sciences, Lanzhou 730046, China; zouyangdr@163.com; 3 Research Center for Parasites & Vectors, College of Veterinary Medicine, Hunan Agricultural University, Changsha 410128, China; 4 College of Veterinary Medicine, Shanxi Agricultural University, Jinzhong 030801, China; 5 State Key Laboratory of Conservation and Utilization of Bio-Resources in Yunnan, Center for Life Science, School of Life Sciences, Yunnan University, Kunming 650500, China; lizhao@ynu.edu.cn

**Keywords:** *Enterocytozoon bieneusi*, prevalence, multi-locus genotype, black goat, Yunnan Province, zoonotic potential

## Abstract

**Simple Summary:**

*Enterocytozoon bieneusi* is one of the most common parasites in human and animals, and a threat to public health. So far, no data are available for *E. bieneusi* prevalence and genotypes in black goats in Yunnan Province, Southwestern China. Therefore, the objective of this study was to detect the prevalence and genotypes of *E. bieneusi* by examining 907 fecal samples collected from 5 counties in Yunnan Province. Ninety-three fecal samples (10.3%) were *E. bieneusi*-positive by PCR amplification. Four new genotypes and 11 known genotypes were identified, and all genotypes considered to be the zoonotic potential. Phylogenetic analysis showed that all of these genotypes were allocated into the zoonotic groups of *E. bieneusi* indicating its zoonotic potential. These results indicated that effective strategies and measures must be taken to prevent and control *E. bieneusi* transmission to other animals and humans.

**Abstract:**

*Enterocytozoon bieneusi* is a fungus-like protist that can parasitize in the intestines of humans and various animals causing a threat to public health. However, there has been no data for *E. bieneusi* prevalence and genotypes in black goats in Yunnan Province, Southwestern China. In this study, 907 fecal samples were collected from black goats in 5 counties from Yunnan Province. The prevalence and genotypes of *E. bieneusi* were examined by nested PCR amplification targeting the nuclear internal transcribed spacer (ITS). Multi-locus sequence typing (MLST) was used to further examine the potential occurrence of genetic segregation. The overall prevalence of *E. bieneusi* in black goats in Yunnan Province was 10.3% (93/907). Statistical analysis revealed that *E. bieneusi* prevalence was significantly associated with the region, age and gender of black goats (*p* < 0.001). Four new genotypes (CYG-1, CYG-2, CYG-3, CYG-4) and 11 known genotypes (CHG1, CHG2, CHG3, CHG5, CHG28, J, D, BEB6, Wildboar3, CD6, SDD1) of *E. bieneusi* were identified. At the microsatellite and minisatellite loci, 15, 2, 17, and 33 sequences were obtained, respectively, forming one new multi-locus genotype (MLG27). Phylogenetic analysis revealed that all 15 genotypes were clustered into group 1 and group 2, with zoonotic potential. This is the first report of *E. bieneusi* prevalence and genotypes in black goats in Yunnan Province, China. Effective control strategies and measures should be taken to reduce the risk of *E. bieneusi* transmission between black goats, other animals, and humans.

## 1. Introduction

Microsporidia comprises a highly diverse group of obligate intracellular parasites, and more than 220 genera and approximately 1700 species have been reported [1]. Most of them could adapt well to the specific hosts but except some species [2]. Among them, 17 species have been reported to cause human microsporidiosis [3], and *Enterocytozoon bieneusi* is the major species infecting humans and many animals. *E. bieneusi* can be transmitted by a fecal–oral route, via contaminated water, food, and directly ingested [4]. After infection with *E. bieneusi*, microsporidiosis is usually asymptomatic, but in immunosuppressed individuals it can cause diarrhea, vomiting, and intestinal cramps, and in severe cases, it will cause death [3,5]. It has been reported that young animals are more susceptible to *E. bieneusi* causing symptoms of diarrhea, weight loss, and even death [6]. The diagnosis of intestinal *E. bieneusi* and other microsporidian species cannot be made easily by microscopic methods because of their small sizes and morphology of their spores. Polymerase chain reaction (PCR) is the best approach to identify *E. bieneusi* by amplification of DNA from the stool sample [7].

To date, more than 500 genotypes have been described and these genotypes have been allocated in 11 phylogenetically major groups. Group 1 and group 2 contain the zoonotic genotypes of *E. bieneusi* [6]. At present, BEB6 is the major genotype of *E. bieneusi* that has been identified from goats in Henan Province [8,9], Yunnan Province [9,10], Heilongjiang Province [11], and Ningxia Hui Autonomous Region [12,13]. CHG1 is the prevalent genotype of *E. bieneusi* in goats in Shaanxi Province [8]. The other genotypes including CHG 1-10, J, D, CD6, COS-1, and CHG15-20 have been identified in farmed and wild goats in China [14].

The black goat (*Capra hircus*) is an important livestock species in Yunnan Province, and it is mainly distributed in mountainous areas, hilly areas, and mountainous areas with more bushes [15]. However, there has been no information on the prevalence and genotypes of *E. bieneusi* in black goats in Yunnan Province, Southwestern China. Hence, the present study investigated the prevalence and molecular diversity of *E. bieneusi* in black goats in Yunnan Province, Southwestern China using PCR-based approaches.

## 2. Materials and Methods

### 2.1. Samples Collection and Preparation

With the permission of farm owners or managers, a total of 907 fecal samples from black goats were collected from Wuding County (25°53′ N, 102°41′ E), Mouding County (25°32′ N, 101°54′ E), Yongren County (26°06′ N, 101°67′ E), Ninglang County (27°28′ N, 100°85′ E), and Mengla County (21°48′ N, 101°56′ E) in Yunnan Province, Southwestern China from August to September, 2017. The fecal samples were put into the 50-mL tube with 2.5% potassium dichromate. The fecal samples were encased with ice pack and transported to the laboratory. All samples were stored at −20 °C freezer until DNA extraction. The information of each sample such as region, age, and gender were recorded.

### 2.2. Genomic DNA Extraction

Prior to extraction of the genomic DNA from fecal samples, each fecal sample was washed by ultrapure water until all potassium dichromate was washed off. Approximately 200 mg of each fecal sample were transmitted into 2 mL centrifuge tube for extraction of genomic DNA using the commercial E.Z.N.A.^®^ Stool DNA kit (Omega Bio-Tek Inc., Norcross, GA, USA) by following the manufacturer’s protocols. Then, the DNA samples were stored at −20 °C until PCR amplification.

### 2.3. PCR Amplification and Sequencing

In this study, all DNA samples were screened by PCR amplification of the nuclear internal transcribed spacer (ITS) region of *E. bieneusi* [16], and three microsatellite loci and one minisatellite locus [17] were used to further identify the multi-loci genotype. All primers used in this study are listed in Table S1. The PCR reaction mixture (25 µL) contained 2 µL of template, 2 µL of dNTP mixture, 2.5 µL of 10 × PCR buffer (Mg^2+^ free), 1.5 mM of MgCl_2_, 0.2 µM of each primer, and 0.625 U of r-*Taq* DNA polymerase (TaRaKa Bio Inc., Tokyo, Japan). The conditions and cycling parameters of the primary PCR and nested PCR were performed as follows: initial denaturation for 1 min at 94 °C, followed by 35 cycles of 45 s at 94 °C for denaturation, 45 s at 55 °C for annealing, 1 min at 72 °C for extension, and a final extension at 72 °C for 10 min [12]. Positive and negative control samples were added to each PCR amplification to ensure the reliability of the result. The secondary amplification products were examined using 1.2% agarose gels with ethidium bromide (EB). All positive products were sent to Xi’an Qingk Biotechnology Company (Xi’an City, China) for two-directional sequencing on an ABI PRISM3730 XL DNA Analyzer (Applied Biosystems, Foster City, CA, USA) using relevant internal primers for PCR amplification.

The obtained sequences were aligned with the relevant sequences available in GenBank database (http://www.ncbi.nlm.nih.gov/GenBank, accessed on 13 August 2021) using Basic Local Alignment Search Tool (BLAST) and Clustal X 1.83 to determine the genotypes of *E. bieneusi*. The known and new genotypes of *E. bieneusi* were identified accordingly to the genotype nomenclature based on the ITS sequences [18]. All samples with new genotypes were sequenced twice to ensure the reliability of the data.

The representative ITS sequences, microsatellite, and minisatellite sequences were deposited in GenBank database, the accession numbers for ITS sequences are MZ479291 to MZ479305, for MS1 are MZ494462 to MZ494468, for MS3 is MZ494469, for MS4 are MZ494470 to MZ494472, and for MS7 are MZ494473 to MZ494475.

### 2.4. Phylogenetic Analysis

The *E. bieneusi* ITS sequences from the present study and the representative sequences from previous studies were used to construct the phylogenetic tree using the Neighbor-joining (NJ) method in MEGA7 [19]. The Kimura 2-parameter model was selected to calculate the genetic distances and bootstrap value was set to 1000 replicates [6].

### 2.5. Statistical Analysis

The prevalence and risk factors of *E. bieneusi* infection were analyzed using the χ2 test in SPSS 24.0 (SPSS Inc., Chicago, IL, USA), and statistically significant differences were considered when *p* < 0.05. Odds ratios (ORs) and 95% confidence intervals (95% CIs) were estimated to explore the strength of the association between *E. bieneusi* infection and the investigated conditions.

## 3. Results

### 3.1. Prevalence of E. bieneusi in Black Goats

In this study, we screened all fecal samples of black goats for *E. bieneusi* positivity by PCR amplification of the nuclear ITS region. Of 907 black goat fecal samples, 93 (10.3%, 95% CI: 8.3–12.2) were *E. bieneusi*-positive (Table 1). The prevalence of *E. bieneusi* ranged from 0 to 14.6% among the five sampled counties. The highest prevalence was 14.6% in Wuding County, but no positive samples were detected in the Mengla County. Female black goats (22.3%, 61/274) were more susceptible to infection with *E. bieneusi* than the males (5.1%, 32/633). Among age groups, the lambs (age < 12 months) (19.3%, 41/212) had the highest *E. bieneusi* prevalence, followed by adult black goats (6.3%, 29/463, age ≥ 24 months) and young black goats (9.9%, 23/232, 12 ≤ age < 24 months). The region, age, and gender factors were all significantly related to *E. bieneusi* prevalence in black goats (*p* < 0.001).

### 3.2. Analysis of E. bieneusi Genotype and MLGs

A total of 15 ITS genotypes of *E. bieneusi* were identified, including 11 known genotypes (CHG1, CHG2, CHG3, CHG5, CHG28, J, D, BEB6, Wildboar3, CD6, SDD1) and 4 new genotypes (designated CYG-1, CYG-2, CYG-3, CYG-4). Among 93 positive samples, CHG1 (29.0%, 27/93) was the dominant genotype in black goats, but it was only detected in Wuding county (Table 2). The infection rate of *E. bieneusi* in black goats in Farm 9 and Farm 15 is 0, which may be related to the breeding management, geographic and ecological factors. The genotype Wildboar3 (17.2%, 16/93) was the second most prevalent genotype, followed by the genotype BEB6 (16.1%, 15/93). Genotypes Wildboar3 and BEB6 were widely distributed among all 12 farms. In addition, genotypes J, D, CD6, CHG2, CHG3, CHG5, CHG28, and SDD1 were also detected in this study.

Sequence analysis revealed that the novel genotype CYG-2 (MZ479293) showed a 99.59% similarity with one single nucleotide polymorphism (SNP) to the genotype BEB6 (MH794170). Novel genotypes CYG-1 (MZ479291), CYG-3 (MZ479298), and CYG-4 (MZ479290) showed 98.35% similarity to the genotype D (AF101200) with 4, 4, and 4 SNPs at different nucleotide positions, respectively (Table 3).

To further explore the genetic variation of *E. bieneusi* in black goats in Yunnan Province, the three microsatellite (MS1, MS3, MS7) loci and one minisatellite (MS4) locus were amplified from all *E. bieneusi*-positive samples. Among all *E. bieneusi*-positive samples, the amplification efficiency of four loci (MS1, MS3, MS4, and MS7) was 22.6% (21/93), 2.6% (2/93), 17.2% (16/93), and 34.4% (32/93), respectively. At four loci, 7, 1, 3, and 3 haplotypes were obtained through sequence analyses, respectively. Only two samples were successfully amplified at all of the four loci, forming one MLG (named MLG27), while others were only successfully amplified at three genetic loci (MS1, MS4, and MS7) (Table 4).

### 3.3. Phylogenetic Analysis of E. bieneusi in Black Goats

**Figure 1:**
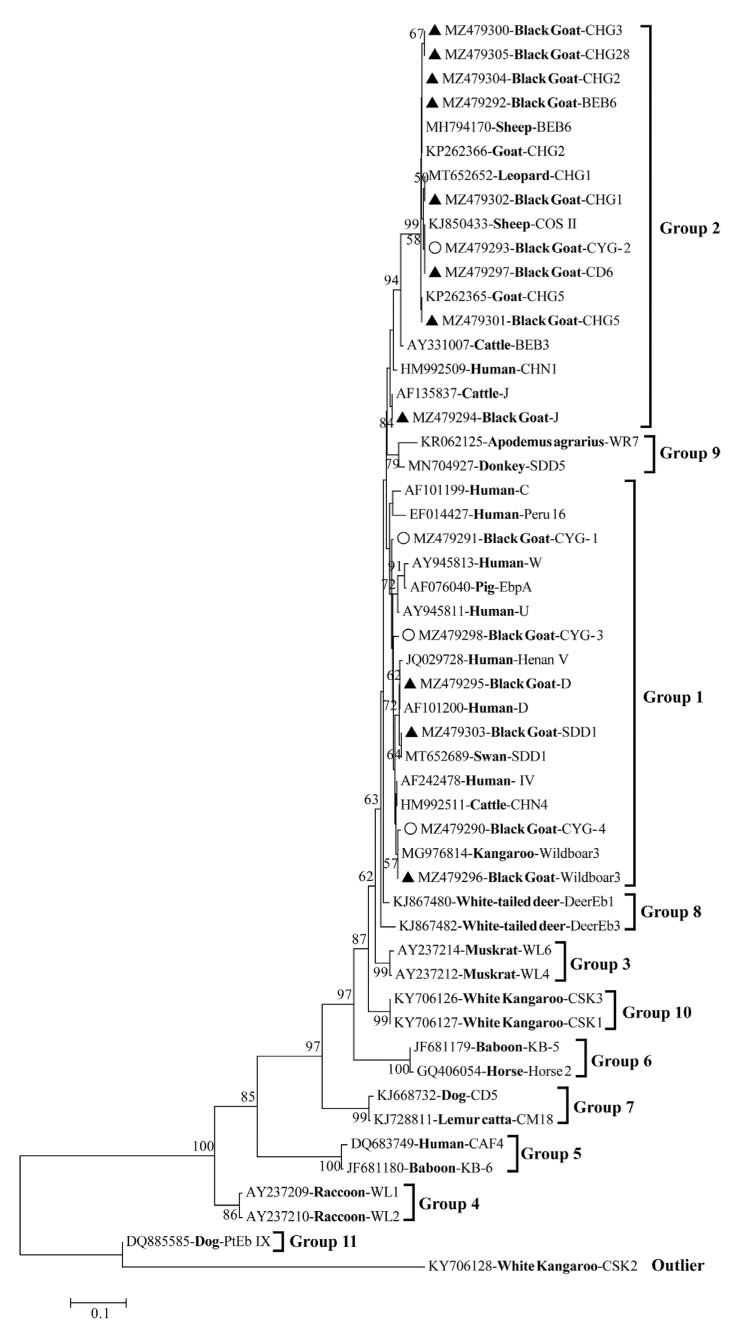
Phylogenetic tree of *Enterocytozoon bieneusi* genotypes based on ITS sequences. All the known genotypes and new genotypes identified in this study were marked by black triangles and unfilled circles, respectively. Bootstrap value is shown when value over than 50%.

Phylogenetic tree was used to evaluate the genetic relationship of 15 genotypes (11 known genotypes and 4 new genotypes) of *E. bieneusi*. Three known genotypes (D, Wildboar3 and SDD1) and three new genotypes (CYG-1, CYG-2, and CYG-3) were clustered into group 1, and eight known genotypes (BEB6, J, CHG1, CHG2, CHG3, CHG5, CHG28, and CD6) and one new genotype (CYG-2) were clustered into group 2 (Figure 1).

## 4. Discussion

*E. bieneusi* infection has been constantly reported in goats in China and other countries (Table 5), and the *E. bieneusi* prevalence in goats ranges from 0 % to 73.6% in the world (Table 5). In this study, the *E. bieneusi* prevalence in black goats in Yunnan Province, Southwestern China was 10.3% (93/907), which was much lower than that in goats in Egypt (100%, 11/11) [20], Thailand (19.2%, 14/73) [21], Spain (14.2%, 1/7) [22], and Henan Province (73.6%, 106/144) [8], Chongqing City (62.5%, 5/8) [9], Shaanxi Province (47.8%, 22/46, and 43.5%, 74/170) [8,9], Henan Province (32.9%, 113/343) [9], Ningxia Hui Autonomous Region (29.7%, 89/300) [13], Hainai Province (24.0%, 82/341) [23], Yunnan Province (22.4%, 30/134) [9], Qinghai Province (18.6%, 11/59) [24], and Heilongjiang Province (21.8, 12/55) [11]; but it is higher than that in goats in Slovakia (0%, 0/20) [25], the Tibet Autonomous Region (9.6%, 25/260) [26], Yunnan Province (8.93%, 30/336) [10], Anhui Province (5.2%, 30/574, and 7.5%, 6/80) [9,27], Jiangsu Province (2.7%, 2/74) [27], Shandong Province (0%, 0/24), and Henan Province (0%, 0/109) [27]. The reason for the different prevalence is complicated, and many factors will affect the detection rate such as sampling time, age group, sampling number and geographic conditions. The *E. bieneusi* prevalence was significantly different among different areas (*p* < 0.001), different age groups (*p* < 0.001), and different genders (*p* < 0.001) in this study, which are consistent with some previous studies [8,9,10]. The relationship between higher prevalence and young age may be associated with immune status of lambs.

In the present study, 11 known and 4 new genotypes of *E. bieneusi* were identified in black goats in Yunnan Province, Southwestern China. The principal genotype was CHG1 (29.0%, 27/93). As a common genotype found in livestock animals, CHG1 has also been detected in non-human primates (NHPs), goats, and wild animals [14,28], which suggests a risk of cross-species transmission. The genotype BEB6 is the predominant genotype of *E. bieneusi* in goats in many reported studies [9,10]. In addition, BEB6 has been reported in humans [29,30], NHPs [28], domestic animals [8,9], wild animals [31], companion animals and birds [32]. This finding suggests that black goats could be a reservoir of *E. bieneusi* to infect humans and other animals. The genotype Wildboar3 was firstly reported in black goats in this study, and previously it was detected in wild animals, such as fox (*vulpes lagopus*) in China [33] and raccoon dog in China [34]. Furthermore, the genotypes J and D were identified in black goats, these two genotypes were found frequently in humans [6], and their zoonotic potential have been confirmed [29,35,36]. The genotypes CD6, CHG3, and CHG5 have been frequently identified from farmed sheep and goats in China [9], pet cat, stray and pet dogs in China [32], and farmed cattle in China [37].

Multi-locus sequence typing (MLST) is a simple and powerful tool to explore the transmission patterns of *E. bieneusi* [38]. MLST is usually used to analyze the potential occurrence of genetic segregation. MLST tool of *E. bieneusi* includes three microsatellites (MS1, MS3, and MS7) and one minisatellite (MS4). Due to the poor amplification efficiency in *E. bieneusi* isolates from groups 2 to 11 by MLST tool [39], in this study, only one MLG was composed and named MLG27 based on CHG1 genotype. This phenomenon was also verified by other works [10,40,41,42], the possible reason is that hypermutations in genomes prevent the amplification of some locus [6]. More reliable genetic markers need to be amplified in further studies to better understand the genetic characteristics and host specificity of *E. bieneusi*.

Phylogenetic analysis of *E. bieneusi* can explore the genetic relationship of genotypes in host specific groups (group 3–11) and zoonotic groups (group 1, 2). The *E. bieneusi* group 1 and group 2 also have a wide host range. As shown in Figure 1, three new genotypes (CYG-1, CYG-3, CYG-4) were clustered to group 1, and one new genotype (CYG-2) was clustered to group 2. These results revealed that the known and novel genotypes found in this study have zoonotic potential and can be transmitted to humans and other animals.

## 5. Conclusions

The present study revealed 10.3% *E. bieneusi* prevalence in black goats in Yunnan Province, Southwestern China. Region, gender and age were significantly associated with the prevalence of *E. bieneusi*. CHG1 was the preponderant genotype, and Wildboar3 and SDD1 were firstly identified in black goats. The four new (CYG-1, CYG-2, CYG-3, CYG-4) genotypes have zoonotic potential. These findings have important implications for the control and prevention of *E. bieneusi* infection in black goats.

## Figures and Tables

**Table 1:** Prevalence and risk factors of *Enterocytozoon bieneusi* infection in Yunnan black goats.

Factor	Categories	No. Tested	No. Positive	Prevalence% (95% CI)	OR	*p*-Value
Area	Wuding	444	65	14.6 (11.4–17.9)	8.6 (1.2–63.2)	*p* < 0.001
	Mouding	145	13	9.0 (4.3–13.6)	4.9 (0.6–38.6)	
	Yongreng	139	14	10.1 (5.1–15.1)	5.6 (0.7–43.7)	
	Ninglang	51	1	2.0 (−1.8–5.8)	Ref.	
	Mengla	128	0	0	-	
Gender	Female	274	61	22.3 (17.3–27.1)	5.4 (3.4–8.5)	*p* < 0.001
	Male	633	32	5.1 (3.3–6.8)	Ref.	
Age	M < 12	212	41	19.3 (14.0–24.7)	3.6 (2.6–6.0)	*p* < 0.001
	12 ≤ M < 24	232	23	9.9 (6.1–13.8)	1.6 (0.9–2.9)	
	M ≥ 24	463	29	6.3 (4.1–8.5)	Ref.	
Total		907	93			

**Table 2:** Genotypes of *Enterocytozoon bieneusi* identified in Yunnan black goats in different farms.

Region	Farm ID	No. Positive/Total	Prevalence (%)	Genotype
Wuding	Farm 1	0/8	0	-
	Farm 2	2/99	2.0	CYG-3 (1), CHG3 (1)
	Farm 3	18/127	14.2	Wildboar3 (8), CHG5 (7), CHG1 (2), D (1)
	Farm 4	27/50	54.0	CHG1 (23), D (1), Wildboar3 (1), CD6 (1), SDD1 (1)
	Farm 5	2/30	6.7	BEB6 (1), CHG3 (1)
	Farm 6	4/23	17.4	CHG3 (2), BEB6 (1), CHG2 (1)
	Farm 7	7/42	16.7	BEB6 (2), CHG1 (2), CHG28 (2), Wildboar3 (1)
	Farm 8	5/35	14.3	CHG3 (5)
	Farm 9	0/30	0	-
Mouding	Farm 10	8/81	11.1	BEB6 (4), CYG -3 (1), CYG -4 (1), D (1), Wildboar3 (1)
	Farm 11	6/64	7.8	BEB6 (2), Wildboar3 (2), CYG-1 (1), CHG3 (1)
Yongreng	Farm 12	6/89	6.7	BEB6 (3), Wildboar3 (2), CHG3 (1)
	Farm 13	7/50	14.0	CYG -2 (2), BEB6 (2), J (2), Wildboar3 (1)
Ninglang	Farm 14	1/51	2.0	CD6 (1)
Mengla	Farm 15	0/128	0	-
Total		93/907	10.3	CHG1 (27), Wildboar3 (16), BEB6 (15), CHG3 (11), CHG5 (7), D (3), J (2), CD6 (2), CHG28 (2), CYG-2 (2), CYG-3 (2), CHG2 (1), SDD1 (1), CYG-1 (1), CYG-4 (1)

**Table 3:** Genetic polymorphisms in the ITS region of novel genotypes of *Enterocytozoon bieneusi* isolates from black goats in this study.

Genotype	Nucleotide Position							GenBank ID
	33							
BEB6	T							MH794170
CYG-2	C							MZ479293
	32	34	82	94	114	118	132	
D	G	C	C	C	C	T	G	AF101200
CYG-1	A	C	T	T	C	G	G	MZ479291
CYG-3	G	T	C	T	T	T	A	MZ479298
CYG-4	A	C	C	T	C	G	A	MZ479290

**Table 4:** Multi-locus characterization of *Enterocytozoon bieneusi* in black goats in Yunnan Province.

ITS Genotype	Multi-Locus Genotypes				MLGs (No.)
	MS1	MS3	MS4	MS7	
CHG1	Type19	Type1	Type14	Type12	MLG27 (2)
CHG1	Type19	-	Type14	Type12	-
CHG1	Type18	-	Type14	Type12	-
CHG1	Type20	-	Type15	Type13	-
CHG1	Type20	-	Type14	Type14	-
CHG3	Type18	-	Type14	Type13	-
CHG3	Type21	-	Type16	Type13	-
CHG1	Type22	-	-	Type12	-
CHG1	Type19	-	-	Type12	-
CHG1	Type24	-	-	Type12	-
CD6	Type18	-	-	Type12	
CHG1	Type23	-	-	-	
CYG-3	Type23	-	-	-	-

**Table 5:** Prevalence and genotypes of *Enterocytozoon bieneusi* in goats in the world.

Country	County	No. of Positive/Tested	Prevalence%	Gene Locus	ITS Genotypes	References
Thailand		14/73	19.2	ITS	Unknown	[21]
China	Ningxia	89/300	29.7	ITS	Unknown	[13]
	Anhui	30/574	5.2	ITS	CHG3 (14), CHG1 (12), AHG2 (2). AHG1 (1), COS-Ⅱ (1)	[27]
	Jiangsu	2/74	2.7	ITS	CHG3 (1), BEB6 (1)	
	Shandong	0/24	0	ITS	-	
	Henan	0/109	0	ITS	-	
	Henan	113/343	32.9	ITS	BEB6 (22), CHG1 (13), CHG3 (10), CHG2 (6), CD6 (4), E (3), CHG5 (3), F (2), KIN-1 (2), D (2), J (1), COS-I (1), CHG6 (1), CHG7 (1), CHG8 (1), CHG9 (1), CHG10 (1), CHG11 (1), CHG13 (1), CHG18 (1), CHG20 (1), CHG21 (1), CHG22 (1), CHG23 (1), CHG25 (1)	[9]
	Yunnan	30/134	22.4	ITS	BEB6 (14), E (4), F (1), CHG3 (2), D (1), COS-I (1), CD6 (1), CHG1 (1), CHG5 (1), CHG16 (1), CHG17 (1), CHG19 (1)	
	Anhui	6/80	7.5	ITS	BEB6 (1), CHG5 (1), CHG3 (1)	
	Chongqing	5/8	62.5	ITS	CHG1 (2), CHG3 (1), CD6 (1), CHG12 (1)	
	Shaanxi	22/46	47.8	ITS	BEB6 (4), CHG1 (3), CHG3 (3), CD6 (3), CHG5 (2), E (1), F (1), CHG14 (1), CHG16 (1), CHG24 (1)	
	Hainai	82/341	24.0	ITS	CHG5 (47), CHG3 (23), CHG2 (4), CM21 (3), D (2), AHG1 (1), HNG-I (1), HNG-II (1)	[23]
	Tibet	25/260	9.6	ITS	EbpA (15), EbpC (16)	[26]
	Yunnan	30/336	8.93	ITS	BEB6 (10), COS-Ⅰ (4), SX1 (3), CM21 (2), CHG3 (2), PigEb4 (1), CHS5 (1), EbpC (1), YNS1 (1)	[10]
	Heilongjiang	12/55	21.8	ITS	Peru6 (3); BeB6 (3); D (2); EbpC (2); EbpA (1); COG-I (1)	[11]
	Shaanxi	179/629	28.5	ITS	SX1 (56), CHG1 (42), BEB6 (6), CHG2 (2)	[8]
	Henan	106/144	73.6	ITS	BEB6 (45), COS-I (14), CHS7 (14)	
	Qinghai	11/59	18.6	ITS	CHG (9), CHG3 (2)	[24]
Egypt	Giza	11/83	13.3	SSU rRNA	Unknown	[20]
Spain		1/7	14.2	SSU rRNA	Unknown	[22]
Slovakia		0/20	0	ITS SSU rRNA	Unknown	[25]

## Data Availability

The data sets supporting the results of this article have been submitted to the GenBank and accession number shown in the article.

## Supplementary Materials

The following are available online at https://www.mdpi.com/article/10.3390/ani11123387/s1, Table S1: The primers of *Enterocytozoon bieneusi* used in this study.

## References

[1] Han B., Pan G., Weiss L.M. (2021). Microsporidiosis in humans. Clin. Microbiol. Rev..

[2] Stentiford G.D., Becnel J., Weiss L.M., Keeling P.J., Didier E.S., Williams B.P., Bjornson S., Kent M.L., Freeman M.A., Brown M.J.F. (2016). Microsporidia—Emergent pathogens in the global food chain. Trends Parasitol..

[3] Han B., Weiss L.M. (2018). Therapeutic targets for the treatment of microsporidiosis in humans. Expert Opin. Ther. Targets.

[4] Santin M., Fayer R. (2011). Microsporidiosis: *Enterocytozoon bieneusi* in domesticated and wild animals. Res. Vet. Sci..

[5] Didier E.S., Weiss L.M. (2006). Microsporidiosis: Current status. Curr. Opin. Infect. Dis..

[6] Li W., Feng Y., Santin M. (2019). Host specificity of *Enterocytozoon bieneusi* and public health implications. Trends Parasitol..

[7] Garcia L.S. (2002). Laboratory identification of the microsporidia. J. Clin. Microbiol..

[8] Peng X.Q., Tian G.R., Ren G.J., Yu Z.Q., Lok J.B., Zhang L.X., Wang X.T., Song J.K., Zhao G.H. (2016). Infection rate of *Giardia duodenalis*, *Cryptosporidium* spp. and *Enterocytozoon bieneusi* in cashmere, dairy and meat goats in China. Infect. Genet. Evol..

[9] Shi K., Li M., Wang X., Li J., Karim M.R., Wang R., Zhang L., Jian F., Ning C. (2016). Molecular survey of *Enterocytozoon bieneusi* in sheep and goats in China. Parasit. Vectors.

[10] Chen D., Wang S.S., Zou Y., Li Z., Xie S.C., Shi L.Q., Zou F.C., Zhu X.Q., Yang J.F., Zhao G.H. (2018). Prevalence and multi-locus genotypes of *Enterocytozoon bieneusi* in black-boned sheep and goats in Yunnan Province, southwestern China. Infect. Genet. Evol..

[11] Zhao W., Zhang W., Yang D., Zhang L., Wang R., Liu A. (2015). Prevalence of *Enterocytozoon bieneusi* and genetic diversity of ITS genotypes in sheep and goats in China. Infect. Genet. Evol..

[12] Peng J.J., Zou Y., Li Z.X., Liang Q.L., Song H.Y., Li T.S., Ma Y.Y., Zhu X.Q., Zhou D.H. (2019). Occurrence of *Enterocytozoon bieneusi* in Chinese Tan sheep in the Ningxia Hui Autonomous Region, China. Parasitol. Res..

[13] Zhang Y., Mi R., Yang J., Wang J., Gong H., Huang Y., Wang X., Han X., Zhou H., Chen Z. (2020). *Enterocytozoon bieneusi* genotypes in farmed goats and sheep in Ningxia, China. Infect. Genet. Evol..

[14] Wang S.S., Wang R.J., Fan X.C., Liu T.L., Zhang L.X., Zhao G.H. (2018). Prevalence and genotypes of *Enterocytozoon bieneusi* in China. Acta Trop..

[15] Wang D., Zhou L., Zhou H., Hou G., Shi L., Li M., Huang X., Guan S. (2015). Effects of nutritional level of concentrate-based diets on meat quality and expression levels of genes related to meat quality in Hainan black goats. Anim. Sci. J..

[16] Sulaiman I.M., Fayer R., Lal A.A., Trout J.M., Schaefer F.W., Xiao L. (2003). Molecular characterization of microsporidia indicates that wild mammals Harbor host-adapted *Enterocytozoon* spp. as well as human-pathogenic *Enterocytozoon bieneusi*. Appl. Environ. Microbiol..

[17] Feng Y., Li N., Dearen T., Lobo M.L., Matos O., Cama V., Xiao L. (2011). Development of a multilocus sequence typing tool for high-resolution genotyping of *Enterocytozoon bieneusi*. Appl. Environ. Microbiol..

[18] Santin M., Fayer R. (2009). *Enterocytozoon bieneusi* genotype nomenclature based on the internal transcribed spacer sequence: A consensus. J. Eukaryot. Microbiol..

[19] Kumar S., Stecher G., Tamura K. (2016). MEGA7: Molecular evolutionary genetics analysis version 7.0 for bigger datasets. Mol. Biol. Evol..

[20] Al-Herrawy A.Z., Gad M.A. (2016). Microsporidial spores in fecal samples of some domesticated animals living in Giza, Egypt. Iran J. Parasitol..

[21] Udonsom R., Prasertbun R., Mahittikorn A., Chiabchalard R., Sutthikornchai C., Palasuwan A., Popruk S. (2019). Identification of *Enterocytozoon bieneusi* in goats and cattle in Thailand. BMC Vet. Res..

[22] Lores B., del Aguila C., Arias C. (2002). *Enterocytozoon bieneusi* (microsporidia) in faecal samples from domestic animals from Galicia, Spain. Mem. Inst. Oswaldo. Cruz..

[23] Zhou H.H., Zheng X.L., Ma T.M., Qi M., Cao Z.X., Chao Z., Wei L.M., Liu Q.W., Sun R.P., Wang F. (2019). Genotype identification and phylogenetic analysis of *Enterocytozoon bieneusi* in farmed black goats (*Capra hircus*) from China’s Hainan Province. Parasite.

[24] Zhang Q., Zhang Z., Ai S., Wang X., Zhang R., Duan Z. (2019). *Cryptosporidium* spp., *Enterocytozoon bieneusi*, and *Giardia duodenalis* from animal sources in the Qinghai-Tibetan Plateau Area (QTPA) in China. Comp. Immunol. Microbiol. Infect. Dis..

[25] Valencakova A., Danisova O. (2019). Molecular characterization of new genotypes *Enterocytozoon bieneusi* in Slovakia. Acta Trop..

[26] Chang Y., Wang Y., Wu Y., Niu Z., Li J., Zhang S., Wang R., Jian F., Ning C., Zhang L. (2020). Molecular characterization of *Giardia duodenalis* and *Enterocytozoon bieneusi* isolated from Tibetan sheep and Tibetan goats under natural grazing conditions in Tibet. J. Eukaryot. Microbiol..

[27] Li W.C., Wang K., Gu Y.F. (2019). Detection and genotyping study of *Enterocytozoon bieneusi* in sheep and goats in east-central China. Acta Parasitol..

[28] Yu F., Wu Y., Li T., Cao J., Wang J., Hu S., Zhu H., Zhang S., Wang R., Ning C. (2017). High prevalence of *Enterocytozoon bieneusi* zoonotic genotype D in captive golden snub-nosed monkey (*Rhinopithecus roxellanae*) in zoos in China. BMC Vet. Res..

[29] Sak B., Brady D., Pelikanova M., Kvetonova D., Rost M., Kostka M., Tolarová V., Hůzová Z., Kváč M. (2011). Unapparent microsporidial infection among immunocompetent humans in the Czech Republic. J. Clin. Microbiol..

[30] Zhang X., Wang Z., Su Y., Liang X., Sun X., Peng S., Lu H., Jiang N., Yin J., Xiang M. (2011). Identification and genotyping of *Enterocytozoon bieneusi* in China. J. Clin. Microbiol..

[31] Qi M., Luo N., Wang H., Yu F., Wang R., Huang J., Zhang L. (2015). Zoonotic *Cryptosporidium* spp. and *Enterocytozoon bieneusi* in pet chinchillas (*Chinchilla lanigera*) in China. Parasitol. Int..

[32] Karim M.R., Dong H., Yu F., Jian F., Zhang L., Wang R., Zhang S., Rume F.I., Ning C., Xiao L. (2014). Genetic diversity in *Enterocytozoon bieneusi* isolates from dogs and cats in China: Host specificity and public health implications. J. Clin. Microbiol..

[33] Zhang X.X., Cong W., Lou Z.L., Ma J.G., Zheng W.B., Yao Q.X., Zhao Q., Zhu X.Q. (2016). Prevalence, risk factors and multilocus genotyping of *Enterocytozoon bieneusi* in farmed foxes (*Vulpes lagopus*), Northern China. Parasit. Vectors.

[34] Xu C., Ma X., Zhang H., Zhang X.X., Zhao J.P., Ba H.X., Du R., Xing X.M., Wang Q.K., Zhao Q. (2016). Prevalence, risk factors and molecular characterization of *Enterocytozoon bieneusi* in raccoon dogs (*Nyctereutes procyonoides*) in five provinces of Northern China. Acta Trop..

[35] Liu H., Jiang Z., Yuan Z., Yin J., Wang Z., Yu B., Zhou D., Shen Y., Cao J. (2017). Infection by and genotype characteristics of *Enterocytozoon bieneusi* in HIV/AIDS patients from Guangxi Zhuang autonomous region, China. BMC Infect. Dis..

[36] Wang L., Xiao L., Duan L., Ye J., Guo Y., Guo M., Liu L., Feng Y. (2013). Concurrent infections of *Giardia duodenalis*, *Enterocytozoon bieneusi*, and *Clostridium difficile* in children during a cryptosporidiosis outbreak in a pediatric hospital in China. PLoS Negl. Trop. Dis..

[37] Li J., Luo N., Wang C., Qi M., Cao J., Cui Z., Huang J., Wang R., Zhang L. (2016). Occurrence, molecular characterization and predominant genotypes of *Enterocytozoon bieneusi* in dairy cattle in Henan and Ningxia, China. Parasit. Vectors.

[38] Tibayrenc M., Ayala F.J. (2012). Reproductive clonality of pathogens: A perspective on pathogenic viruses, bacteria, fungi, and parasitic protozoa. Proc. Natl. Acad. Sci. USA.

[39] Li W., Feng Y., Zhang L., Xiao L. (2019). Potential impacts of host specificity on zoonotic or interspecies transmission of *Enterocytozoon bieneusi*. Infect. Genet. Evol..

[40] Tang C., Cai M., Wang L., Guo Y., Li N., Feng Y., Xiao L. (2018). Genetic diversity within dominant *Enterocytozoon bieneusi* genotypes in pre-weaned calves. Parasit. Vectors.

[41] Wu Y., Chang Y., Chen Y., Zhang X., Li D., Zheng S., Wang L., Li J., Ning C., Zhang L. (2018). Occurrence and molecular characterization of *Cryptosporidium* spp., *Giardia duodenalis*, and *Enterocytozoon bieneusi* from Tibetan sheep in Gansu, China. Infect. Genet. Evol..

[42] Zou Y., Hou J.L., Li F.C., Zou F.C., Lin R.Q., Ma J.G., Zhang X.X., Zhu X.Q. (2018). Prevalence and genotypes of *Enterocytozoon bieneusi* in pigs in southern China. Infect. Genet. Evol..

