# Supplementary material for: Molecular Detection and Genotyping of Enterocytozoon bieneusi in Black Goats (Capra hircus) in Yunnan Province, Southwestern China
Source: Animals (Basel). 2021 Nov 26;11(12):3387. doi: 10.3390/ani11123387 (PMC8698114; doi:10.3390/ani11123387)
Supplement: Supplementary file 1 [file animals-11-03387-s001.zip › animals-1435991-supplementary.pdf]

Table S1. The primers of *Enterocytozoon bieneusi* used in this study.

Locus	Primers	Sequences (5′→3′)	Length (bp)
ITS	NEBF1	GATGGTCATAGGGATGAAGAGCTT	
	NEBR1	TATGCTTAAGTCCAGGGAG	
	NEBF2	AGGGATGAAGAGCTTCGGCTCTG	390
	NEBR2	AGTGATCCTGTATTAGGGATATT	
MS1	MS1F1	CAAGTTGCAAGTTCAGTGTTTGAA	
	MS1R1	GATGAATATGCATCCATTGATGTT	
	MS1F2	TTGTAAATCGACCAAATGTGCTAT	675
	MS1R2	GGACATAAACCACTAATTAATGTAAC	
MS3	MS3F1	CAAGCACTGTGGTTACTGTT	
	MS3R1	AAGTTAGGGCATTTAATAAAATTA	
	MS3F2	GTTCAAGTAATTGATACCAGTCT	537
	MS3R2	CTCATTGAATCTAAATGTGTATAA	
MS4	MS4F1	GCATATCGTCTCATAGGAACA	
	MS4R1	GTTCATGGTTATTAATTCCAGAA	
	MS4F2	CGAAGTGTACTACATGTCTCT	885
	MS4R2	GGACTTTAATAAGTTACCTATAGT	
MS7	MS7F1	GTTGATCGTCCAGATGGAATT	
	MS7R1	GACTATCAGTATTACTGATTATAT	
	MS7F2	CAATAGTAAAGGAAGATGGTCA	471
	MS7R2	CGTCGCTTTGTTTCATAATCTT	
